# Analyzing Land-Use Change Scenarios for Ecosystem Services and their Trade-Offs in the Ecological Conservation Area in Beijing, China

**DOI:** 10.3390/ijerph17228632

**Published:** 2020-11-20

**Authors:** Zuzheng Li, Xiaoqin Cheng, Hairong Han

**Affiliations:** School of Ecology and Nature Conservation, Beijing Forestry University, Beijing 100083, China; lzz2017@bjfu.edu.cn

**Keywords:** ecosystem services, land-use changes, GeoSOS-FLUS, InVEST

## Abstract

It is generally believed that land-use changes can affect a variety of ecosystem services (ES), but the relationships involved remain unclear due to a lack of systematic knowledge and gaps in data. In order to make rational decisions for land-use planning that is grounded in a systematic understanding of trade-offs between different land-use strategies, it is very important to understand the response mechanisms of various ecosystem services to changes in land-use. Therefore, the objective of our study is to assess the effects of land-use change on six ecosystem services and their trade-offs among the ecosystem services in the ecological conservation area (ECA) in Beijing, China. To do this, we projected future land-use in 2030 under three different scenarios: Business as Usual (BAU), Ecological Protection (ELP), and Rapid Urban Development (RUD), using GeoSOS-FLUS model. Then, we quantified six ecosystem services (carbon storage, soil conservation, water purification, habitat quality, flood regulation, and food production) in response to land-use changes from 2015 to 2030, using a spatially explicit InVEST model. Finally, we illustrated the trade-offs and/or synergistic relationships between each ecosystem service quantified under each of the different scenarios in 2030. Results showed that built-up land is projected to increase by 281.18 km^2^ at the cost of water bodies and cultivated land from 2015 to 2030 under the RUD scenario, while forest land is projected to increase by 152.38 km^2^ under the ELP scenario. The carbon storage, soil conservation, habitat quality, and the sum of ecosystem services (SES) would enrich the highest level under the ELP scenario. Land-use strategies that follow the ELP scenario can better maintain the ecosystem services and sustainable development of natural and social economic systems.

## 1. Introduction

Ecosystem services (ES) are the various benefits that people obtain from ecosystems directly or indirectly, such as food production, wildlife habitat, and climate regulation, which can improve human welfare or maintain global life support systems [1,2]. However, in the past few decades, it is estimated that 60% of global ES and biodiversity have been degraded, affecting the ability of the ecosystem to provide adequate services for current and future scenarios [2]. High demands for food, timber, energy, housing, and other goods and services to meet the demands of more than 7 billion people worldwide—as well as the shifts in land management efforts to enhance some ES but at the cost of reductions in many ES—have resulted in the degradation of global ecosystem services at unprecedented intensities and rates [3]. Among all human activities, land-use and land-cover change (LUCC) is always the significant factor affecting the composition and configuration of ecosystems that leads to a change in the provision of ecosystem services [2,4,5], as some ES are closely related to specific land-use types [6]. For example, the conversion of grasslands, forests, and water areas into croplands and developed areas has led to a substantial increase in the production of food, housing, and other commodities [7]. Therefore, in order to meet the growing global demand, exploring the relationship between land use change and ecosystem services has aroused widespread attention of scientists and decision-makers all over the world.

The impacts of land-use changes on ES differ significantly among varying socio-economic backgrounds and across spatial or temporal scales [8,9,10]. Recent studies have shown that the diversified social demands and the environmental complexity have led to more complex interactions among multiple ES [11,12]. For example, an increase of ES provisions (such as food and fiber) can lead to reductions of regulating services (such as water regulation and carbon sequestration), resulting in a trade-off of one ES for another [13,14]. Yet, in another example, the improvement of human living environments that synergistically includes other ES (e.g., urbanization plans, including afforestation, trash separation, and increasing water area) can improve multiple regulating ES (e.g., air filtration, urban heat sinks, and surface water balance for flood regulation), and positively influence regional climates [15,16]. These examples illustrate the relationships between ES in which the increase in one service may lead to decreases of other services (trade-off), or the co-dependent interactions between multiple services may lead to simultaneous enhancement (synergy). Although trade-offs are deliberative decisions that take place all the time, the relationships between ES are not always clear or are often poorly understood, and, as a result, unexpected influences often arise [10,11]. Thus, reevaluating how trade-offs among ecosystem services respond to land-use change will provide informed decisions for policy makers.

A previous study showed that ES evaluations can be incorporated into land-use management in two ways in which one is used as the target of future land-use planning. For example, [17] used the land-use optimization model FUTURES to assess the influence of urban expansion on multiple ES. The other mode of incorporating ES into land-use planning is through ES assessment, a comparison of multiple land-use schemes under different scenarios, and a final selection of the land-use plan that would provide as many synergies between ES and socio-economic design criteria as possible. For example, [18] predicted the urban expansion and ES dynamics of Beijing by using the LUSD-urban model under several scenarios. However, due to the complexity of assessing many parameters, the complex operational processes required and the uncertainty of future development policies’ existing models are insufficient in their ability to predict the potential impact of LUCCs on multiple ecosystem services and their trade-offs in mountainous and urbanized areas [19]. To address this gap, the GeoSOS-FLUS model was developed to simulate land-use under different scenarios [20]. The GeoSOS-FLUS model is composed of the Markov Chain model and the bottom-up cellular automata (CA) model. The Markov model can predict the LUCC quantities using the probability transition matrix, while the CA model is effective at producing the long-term spatial trajectories of multiple LUCCs with transition rules [21,22,23,24]. More importantly, the GeoSOS-FLUS model is an effective and reproducible tool in analyzing both the causes and consequences of alternative future landscape dynamics by considering various socio-economic and natural environmental driving factors [20]. Therefore, the GeoSOS-FLUS model can simulate both temporal and spatial dimensions of future LUCC under variously designed scenarios.

According to the General Planning of Beijing Municipality (2016–2035), the ecological conservation area (ECA) in Beijing is an important ecological barrier, climate regulator, and water protection area, and is key to ensuring the sustainable development of the capital of China. Mountain ecosystems and related ES in the ECA are particularly vulnerable to the impacts of land-use and climate change, which brings a great challenge to the sustainable supply of ES [25,26,27]. Over the past few decades, the ECA has undergone dramatic social and economic transitions, including economic and population growth, and urbanization. These socio-economic transitions have been accompanied by drastic changes in land-use and land-cover, triggering trade-offs between multiple ES that pose a risk for ES degradation, such as soil and water loss, water pollution, and losses of basic farmland [28]. To mitigate these contradictions, the People’s Government of Beijing Municipality introduced a series of policies, including the “Red Lines for Ecological Protection” and “13th Five-Year Plan of Environmental Protection and Ecological Construction” [27,29]. However, most policies either do not fully consider the complex effects of land-use changes, or only promote ecological protection goals without taking into account future uncertainty and the consequences of human activities under a variety of scenarios. Therefore, the objective of the study is to explore how land-use changes would alter multiple ecosystem services and their trade-offs under three different alternative scenarios in the ECA.

In our study, to effectively evaluate the impacts of land-use change on ES, we linked the GeoSOS-FLUS (Sun Yat-sen University, Guangzhou, China) and InVEST (Integrated Valuation of Ecosystem Service and Tradeoffs) models (Stanford University, Palo Alto, CA, USA), which are both suitable for application under different scenarios [6,8,30]. The GeoSOS-FLUS model was used to quantify the changes in land-use between the years 2000, 2015, and 2030, under three scenarios: (1) Business As Usual (BAU), (2) Rapid Urban Development (RUD), and (3) Ecological Protection (ELP). Then, we used the InVEST model to assess changes in six ES: carbon storage (CS), water purification (WP), flood regulation (FR), habitat quality (HQ), soil conservation (SC), and crop production (CP). We then analyzed the impacts of land-use change on these services and their trade-offs under each alternative future scenario. Based on the above assessment, we propose in this paper corresponding land-use strategies and ecological management measures that can support the sustainability of ES in the ECA.

## 2. Materials and Methods 

### 2.1. Study Area

**Figure 1:**
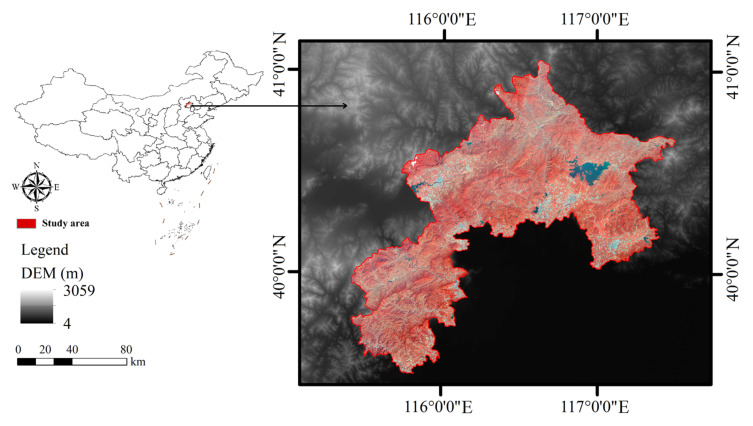
Location of the study area in China, DEM (Digital Elevation Model).

The ecological conservation area (ECA) (41°04′–39°31′ N, 115°24′–117°29′ E) is situated in the western part of Beijing, China (Figure 1). The area covers an area of about 11,140.15 km^2^, belonging to a typical temperate monsoon climate, with altitudes of 11 m to 2304 m and annual precipitation of 576.71 mm. The ecological conservation area (ECA) is characterized by its natural ecological resources [27] with its main ecosystem types including various types of planted forests, water resources, and land used for basic crop cultivation [31]. The ECA contains several national nature reserves, which are the sources of various ecosystem services and the most important areas for ecosystem service protection in Beijing. However, in recent decades, rapid urbanization and economic growth have resulted in habitat degradation and soil erosion, threatening the ECA with frequent natural disasters such as floods.

### 2.2. Data Preparation and Analysis

In our study, land-use maps (30 m spatial resolution) of the ecological conservation area (ECA) in 2000 and 2015 were derived from the Resources and Environmental Sciences, Chinese Academy of Sciences [32]. To finely calculate ecosystem services given the varying availability of data such as carbon density, some types of land-use were subdivided or merged. As a result, seven types of land-use were classified: cultivated land, grassland, forest land, built-up land, water body, shrub land, and unused land (Table S1 in Supplementary Materials). Additionally, the data types used in the InVEST model are shown in Table 1. All the raster data were converted into the Beijing_1954 geographic coordinate system, and the resolution of all raster data were set to 30 m by ArcGIS 10.3 (Environmental Systems Research Institute, Redlands, CA, USA).

### 2.3. Future Scenarios Designing

We developed three alternative future design scenarios: the BAU (Business As Usual) scenario, RUD (Rapid Urban Development) scenario, and ELP (Ecological Protection) scenario by using the GeoSOS-FLUS model [20,39]. We used the land-use map of 2015 as the baseline case scenario for comparison. In the context of climate change, many regions are experiencing a trend toward warming and drought, which increases the possibility of water shortages, natural disasters, and desertification, and has put considerable pressure on the sustainable utilization of ES [40]. However, this study focuses on the changes of ecosystem services caused by land use changes. Therefore, climate data, including temperature and precipitation, were assumed to remain unchanged. Table 2 shows the descriptions of each scenario.

### 2.4. Future Land-Use Modelling

In our study, we used the GeoSOS-FLUS model to predict future scenario land-use maps, which is an integrated LUCC model for multi-type land-use simulation by coupling human and natural influences [20]. The GeoSOS-FLUS model has been widely used throughout the world to facilitate the multiple land use change simulation. It can be divided into two modules: (1) Markov chain, which calculates the area of different land-use types as the non-spatial demand in 2030, and (2) Cellular Automata (CA) allocation model, which was used as a spatially explicit allocation program to estimate spatial patterns [24]. For the Cellular Automata (CA) allocation model, two steps were carried out. First, an artificial neural network (ANN) was used for taking both human activities and natural effects into consideration by finding the complex relationships between land use patterns and various human and natural driving forces. Second, an elaborate self-adaptive inertia and competition mechanism was developed to solve the interactions among multiple land-use types [20,22]. We considered the land-use in 2015 as the reference map for the simulation for 2030, and used the following parameters as inputs of the Cellular Automata (CA) allocation model: (1) restrictions, (2) driving factors, (3) land-use type conversion settings, and (4) predicted land-use areas (demand) from the Markov model. For the Markov chain, we used the land-use of 2015 as the basic map, and then generated the transfer area and probability matrix of land-use types for 2015–2030. We selected fourteen known driving factors of land-use change: (1) Digital Elevation Model (DEM), (2) slope, (3) aspect, (4) relief amplitude, (5) Gross Domestic Product (GDP), (6) annual precipitation, (7) annual mean temperature, (8) distance from road, (9) distance from residential area, (10) distance from traffic station, (11) distance from river, (12) distance from landslide and collapse point, (13) soil attributes, and (14) population density (Supplementary Table S2) [20,41,42]. 

The GeoSOS-FLUS model was implemented to generate the simulation results from 2000 to 2015 and used the Figure of Merit (FoM) to validate the performance of this model. We chose this indicator because it can avoid the drawback of overestimating traditional validation (e.g., the Kappa coefficient) [43,44]. The FoM value ranges from 0 to 1, where 1 represents the perfect model applicability of simulating the land-use map. The FoM value of our GeoSOS-FLUS model was 0.269, which indicates the simulation results were reliable, because the final FoM value is similar to or larger than the application of land-use change modeling [45]. Therefore, these driving factors and parameters are applicable to forecast future LUCC (Table S2). Finally, we ran the GeoSOS-FLUS model to simulate future LUCC in 2030 under three different scenarios.

### 2.5. Ecosystem Services Evaluations

In this study, we used the InVEST 3.8.0 model to quantify the ecosystem services (ES), which was based on the land-use maps of the ECA [46,47]. Since the major ecological problems in the study area are floods, soil erosion, and water resources loss, and the Millennium Ecosystem Assessment identified the needs for key ES such as basic materials for a good life, health, and security [2], we chose to analyze the following ES: crop provision (basic material needs), water purification (health needs for clean water resource), carbon storage (climate regulation), flood regulation, soil conservation, and habitat quality services (safety requirements). The quantification and spatial mapping of ES were conducted at the temporal scale of 2000, 2015, and 2030 under three scenarios, utilizing a series of parameters and data in the InVEST model (Table 1).

#### 2.5.1. Carbon Storage and Sequestration (CS)

The Carbon Storage and Sequestration model was used to estimate the amount of carbon currently stored in an ecosystem or the amount of carbon sequestered over time based on four carbon pools (aboveground biomass, belowground biomass, soil, and dead organic matter) [47] (see Supplementary Table S3). The model aggregated the amount of stored carbon according to the LUCC maps and the carbon density per LUCC type, which were derived from literature based on the local studies [25]. The calculation is as follows (Equation (1)).

*CS_x_* = *Ax* (*Ca_x_* + *Cb_x_* + *Cs_x_* + *Cd_x_*) (1)

where *CS* is the sum of all carbon storage in grid cell *x* (*Mg C ha*^−1^), *Ca* is the carbon density in the aboveground (*Mg C ha*^−1^), *Cb* is the carbon density in the belowground (*Mg C ha*^−1^), *Cs* is the carbon density in the soil (*Mg C ha*^−1^), and *Cd* is the carbon density in the dead matter (*Mg C ha*^−1^).

#### 2.5.2. Flood Regulation (FR)

Flood regulation refers to the ability of a land-use type to intercept or store water resources from precipitation. The annual water yield was used as the inverse indicator to assess flood regulation as follows (Equation (2)).

*FR_x_* = (*Y_max_* − *Y_x_*)/(*Y_max_* − *Y_min_*) (2)

where *FR_x_* is the flood regulation capacity for cell *x*, *Y_x_* is the annual water yield on cell *x*, and *Y_max_* and *Y_min_* are the maximum and minimum water yield from any cells, respectively. The larger water yield produced by the cell *x* indicates a lower capacity of cell *x* to regulate water flow.

The Annual Water Yield model was used to calculate the annual water yield based on pixel *x*. In the model, the vegetation type, plant available water content (%), soil maximum root depth (mm), annual potential evapotranspiration (mm), annual precipitation (mm), and land-use are the main reference factors [47] (Table S4). The water yield is shown as follows (Equation (3)).

*Y_x_* = (1 − *AET_x_*/*P_x_*) · *P_x_* (3)

where *Y_x_* refers to the annual water yield for each grid cell (mm), *AET_x_* is the actual evapotranspiration per grid cell *x* (mm), and *P_x_* is the annual rainfall of each grid cell *x* (mm). *AET_x_*/*P_x_* approximates the Budyko curve developed by [48,49] (Equation (4)).

*AET_x_*/*P_x_* = (1 + *PET_x_*/*P_x_*) − [1 + (*PET_x_*/*P_x_*)*^ω^*]^1/*ω*^ (4)

where *PET_x_* refers to the potential evapotranspiration, and *ω_x_* is a dimensionless ratio that characterizes the natural climatic-soil properties. 

#### 2.5.3. Soil Conservation (SC)

The sediment delivery ratio model was used to map overland sediment generation and delivery to the stream, which was based on the LUCC data, digital elevation model (DEM), soil erodibility (K), rainfall erosivity index (R), and biophysical table containing model information [47,50,51] (Table S5). The formula is as follows (Equations (5)–(7)).

*SC_x_* = *RKLS_x_* − *usle_x_* (5)

*usle_x_* = *R_x_* · *K_x_* ·*LS_x_* · *C_x_* · *P_x_* (6)

*RKLS_x_* = *R_x_* · *K_x_* · *LS_x_* (7)

where *SC_x_* refers to the amount of annual soil retention in cell *x* (*tons*·(*ha*·*yr*)^−1^), *RKLS_x_* is the potential soil erosion without any vegetation coverage and any water conservation practices (*tons*·(*ha*·*yr*)^−1^), and *usle_x_* is the actual soil erosion under the impacts of vegetation coverage considering the soil conservation measures in cell *x* (*tons*·(*ha*·*yr*)^−1^). *R_x_* is the rainfall erosivity factor (*MJ*·*mm*·(*ha*·*h*·*yr*)^−1^), *K_x_* is the soil erodibility factor (*tons ha h* (*ha MJ mm*)^−1^), *LS_x_* is the topographic factor (dimensionless), *C_x_* is a vegetation cover management factor (dimensionless), and *P_x_* is a soil conservation measures factor (dimensionless).

#### 2.5.4. Water Purification (WP)

Water purification was calculated using the “Nutrient Delivery Ratio model” model to measure the contribution of vegetation and soil to water purification by retaining non-point source nutrient pollutants from runoff [47]. The main pollution sources in the ECA is ammonia nitrogen [52]. Therefore, nitrogen (N) export is used to represent the water purification (WP) service. The input data for this model includes maps of land-cover and land-use, runoff, Digital Elevation Model (DEM), soil characteristics, and biophysical attributes related to the nutrient loading and retention efficiency for different LULC types (Table S6). The final nitrogen (kg y^−1^) exports for each grid cell can be calculated using the following equation (Equation (8)).

*ALV_x_* = *HSS_x_* · *pol_x_* (8)

where *ALV*x is the adjusted load value of grid cell *x*, *pol_x_* is the export coefficient at grid cell *x*, and *HSS_x_* is the Hydrologic Sensitivity Score at grid cell *x*, which is calculated as (Equation (9)):

(9) $$HSS_{x}=\frac{\lambda_{x}}{\bar{\lambda_{w}}}$$

where $\lambda_{x}$ is the runoff index at grid cell x, $\bar{\lambda_{x}}$ is the mean runoff index in the watershed, and $\lambda_{x}$ can be calculated using the following equation (Equation (10)).

(10) $$\lambda_{x}=\log(\underset{U}{\sum }Y_{U})$$

where $\sum_{U}Y_{U}$ is the sum of the water yield of grid cells along the flow path above grid cell *x*.

#### 2.5.5. Habitat Quality (HQ)

The “Habitat Quality” module of the InVEST model was used to evaluate the biodiversity of a landscape, which estimates the extent of habitat and vegetation types for organisms across a landscape, and their state of degradation. The model combines information on maps of land-use and land cover (LULC) and threats to biodiversity to produce habitat quality maps. The model assumes that areas with a high-quality habitat will better support all levels of biodiversity, and decreases in habitat extent and quality lead to reductions in biodiversity persistence. Habitat quality is mediated in a grid cell by four factors: (1) the relative impact of each threat, (2) the distance between habitat, the threat source, and the impact of the threat (Tables S7 and S8), (3) the level of legal protection from disturbance, and (4) the relative sensitivity of each habitat type to each threat [47]. The habitat quality in parcel *x* of land-use type *j* can be given by *Q_xj_* and calculated as follows (Equation (11)).

(11) $$Q_{xj}=H_{j}\left(1-D_{xj}\right)$$

where *H_j_* is the habitat suitability of land-use type *j*, and *D_xj_* is the total threat level in grid cell (*x,y*) in land-use type *j*. The value of *Q_xj_* ranges from 0 to 1, where 1 indicates the cell’s habitat quality at its highest level.

#### 2.5.6. Crop Production (CP)

According to the Beijing statistical yearbook [53], cultivated land in Beijing mainly produces three kinds of food: grains, vegetables, and fruits. In this study, grains and vegetables are mainly grown in the plains of each district, while fruits are produced in the mountainous areas, as the distribution of food production in the ECA is mainly determined by topography and availability of irrigation. Therefore, we collected data from the Beijing statistical yearbook for grain, vegetable, and fruit production and distribution in each district. The quantity of crop production under different scenarios can be calculated as follows (Equations (12)–(14)).

(12) $$PRO_{G}=\overset{i}{\underset{i=1}{\sum }}A_{i}\times R_{Gi}\times P_{Gi}$$

(13) $$PRO_{V}=\overset{i}{\underset{i=1}{\sum }}A_{i}\times R_{Vi}\times P_{Vi}$$

(14) $$PRO_{F}=\overset{i}{\underset{i=1}{\sum }}A_{i}\times R_{Fi}\times P_{Fi}$$

where *PRO_G_*, *PRO_V_*, and *PRO_F_* are the production of grains, vegetables, and fruits, respectively. *A_i_* is the area of district *i* in the study area. *R_Gi_*, *R_Vi_*, and *R_Fi_* are the proportions of areas of grains, vegetables, and fruits in district *i*, respectively, and *P_Gi_*, *P_Vi_*, and *P_Fi_* are the yield per unit area for grains, vegetables, and fruits in each district, respectively (ton/ha).

#### 2.5.7. Sum of Ecosystem Services (SES)

A comprehensive indicator of ES can be used to compare the overall level of multifunctional land-use types and their total supply of various ES in different years and scenarios, while a single indicator of ES only reflects the importance of one aspect to a region. In this study, the sum of ecosystem services (SES) indicator was developed to quantify the total supply of multiple ES, according to previous studies [54,55]. The new indicator can spatially reflect the overall condition of ES in space and provide the basis for the government to make urban planning policies. The importance of different ES was determined by different weights. The SES metric is calculated as follows (Equation (15)).

(15) $$SES_{j}=\overset{n}{\underset{i=1}{\sum }}w_{i}\times S_{ij}$$

where *SES_j_* is the sum value of the comprehensive metric for all ecosystem services in the year or the scenario *j*, *w_i_* is the weight assigned to the *i*th ecosystem service, *S_ij_* is the standardized value for the i*th* ecosystem service in the year or the scenario *j*, and *n* is the number of ecosystem services evaluated.

Previous studies have shown that Beijing’s urban development has led to the loss of carbon storage in recent years, which is due to the increase of impervious surface and loss of natural vegetation, threatening the ecological security of the ECA [25,38]. Therefore, the assessment of carbon storage is of great significance for Beijing to adapt to maintain sustainable development. The terrain in this area is characterized by a large mountainous area and large relief, which is vulnerable to geological disasters such as floods, debris flow, and landslides. Soil conservation, nutrient retention, and flood regulation are also considered to be comparatively important ecosystem services in the area. In addition, most basic farmland for fruit and vegetable production are located in this area in Beijing. However, driven by rapid population growth, Beijing’s demand for food has increased dramatically, and more than 90% of grain needs to be imported from other provinces [53]. Therefore, we assigned the weights to ecosystem services as follows: carbon storage (0.1596), flood regulation (0.1688), water purification (0.0875), soil conservation (0.1396), habitat quality (0.1574), and crop provision (0.0964) (see Table 3). These weights are designed to take into account the unique local conditions in Beijing, and makes reference to Sun et al. (2018).

To ensure that different ES can be added together, we standardized the values of ES using two independent formulas in each grid cell. The positive indicators including carbon storage, flood regulation, soil conservation, habitat quality, and crop provision are as follows (Equation (16)).

(16) $$ES_{x}=\frac{E_{x}-E_{min}}{E_{max}-E_{min}}$$

where *ES_x_* is the standardized value (value of 0 to 1) on each pixel *x*, *E_x_* is the original ES value on each pixel *x*, and *E_max_* and *E_min_* are the maximum and minimum value of any pixels, respectively. 

The negative indicator was nitrogen retention that can be expressed as follows (Equation (17)).

(17) $$S_{x}=\frac{E_{max}-E_{x}}{E_{max}-E_{min}}$$

For all standardized value of the six ecosystem services, 1 refers to the best performance and 0 refers to the worst performance.

### 2.6. Calculation of the Trade-Offs of ES

The Spearman’s coefficient was used to examine the trade-off/synergies among ES. First, we created sampling points by using the “Create Random Points tool” in the data management toolbox of the ArcGIS 10.3, and then used the “Extract Multiple Values of Points” method to extract the ecosystem service value of each sampling point. After analysis, 2000 sampling points were selected in this study. Finally, the SPSS statistical software (Version 24.0, International Business Machines Corporation, Armonk, NY, USA) was used to analyze the correlation based on the service value (Pearson, two-tailed) of these points.

## 3. Results

### 3.1. Land-Use Changes Between 2000 and 2030

**Figure 2:**
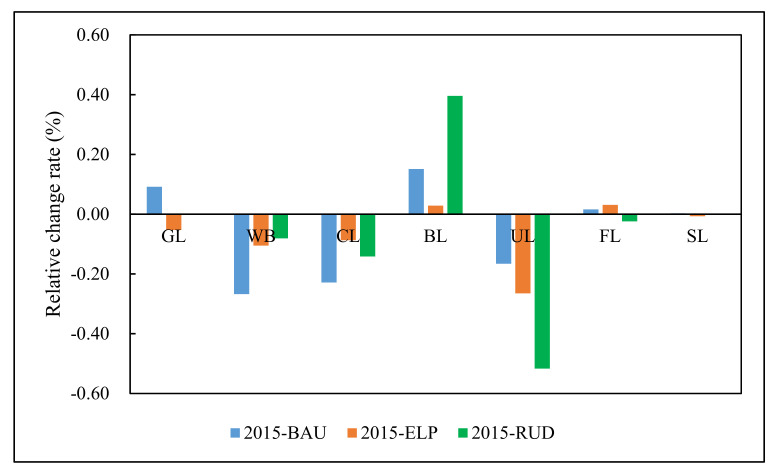
Land-use changes (%) from 2015 to 2030 under three scenarios in the ECA, GL (Grass Land), WB (Water Body), CL (Cultivated Land), BL (Built-up Land), UL (Unused Land), FL (Forest Land), SL (Shrub Land), BAU (Business as Usual), ELP (Ecological Protection), and RUD (Rapid Urban Development).

**Figure 3:**
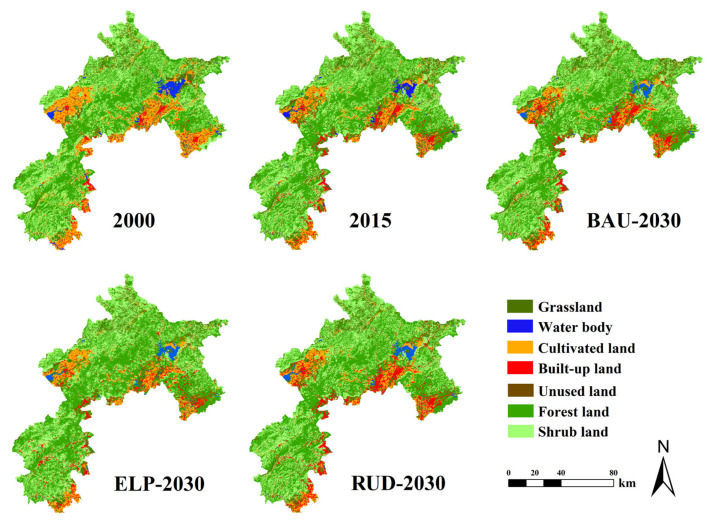
Land-use maps of the ecological conservation area (ECA) from 2000 to 2030 under the Business-As-Usual (BAU) scenario, Ecological Protection (ELP) scenario, and Rapid Urban Development (RUD) scenario.

Throughout the whole study period, forest and shrub land are the two largest land-use types in the ecological conservation area (ECA), and several transitions in land-use types are predicted (Figure 2 and Figure 3). From 2000 to 2015, the areas of grassland, built-up land, forest, and shrub land increased. Among them, the proportion of forest land increased the most by 4.04%, while the areas of water body, cultivated land, and unused land decreased (Tables S9 and S10). Cultivated land decreased the most with 6.80%. From 2015 to 2030, the main characteristic of land-use change is the expansion of built-up land at the expense of cultivated land, water body, and unused land. The extent of built-up land expansion between 2015 and 2030 was predicted to grow by 0.96% and 2.52% under the business as usual (BAU) and rapid urban development (RUD) scenarios, respectively, but remain relatively stable under the ecological protection (ELP) scenario. In contrast, the area of cultivated land was predicted to decrease by 1.92%, 0.73%, and 1.19% under the BAU, ELP, and RUD scenarios, respectively. The forest land was predicted to increase by 77.4 km^2^ (0.69%) and 152.38 km^2^ (1.36%) under the BAU and ELP scenarios, respectively, but decrease by 119.54 km^2^ (−1.08%) under the RUD scenario.

### 3.2. Changes of Ecosystem Services Between 2000 and 2030

**Figure 4:**
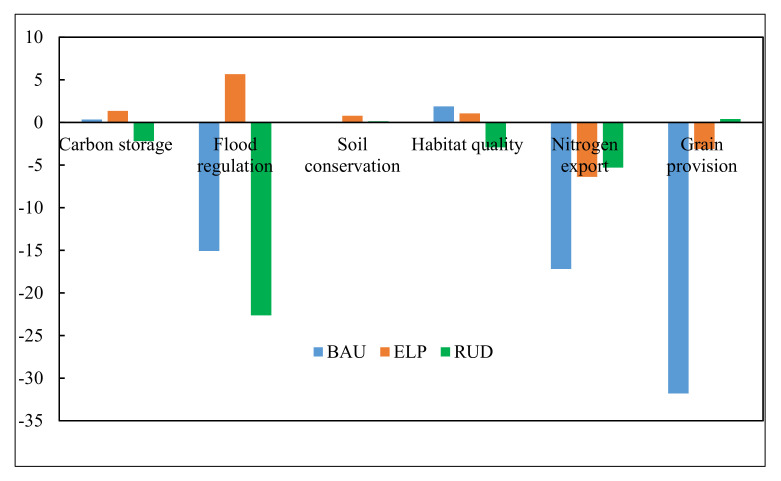
Changes (%) in different ecosystem services in the ECA from 2015 to 2030 under three alternative scenarios.

As shown in Table 4 and Figure 4, from 2000 to 2015, the total carbon storage, flood regulation capacity, and habitat quality increased by 4%, 36%, and 7%, respectively, while soil conservation, water purification, and crop provision decreased by 8%, 27%, and 18%, respectively. These changes were mainly caused by the rapid erosion of cultivated land and an increase of forest land in the past few years. In 2015, the total carbon storage was 0.99 × 10^8^ tons, which was predicted to decrease to 0.97 × 10^8^ tons in 2030 under the RUD scenario, while the highest amount of carbon storage under the ELP scenario is expected (1 × 10^8^ tons). The flood regulation was predicted to increase from 0.53 in 2015 to 0.56 in 2030 under the ELP scenario, but decrease to 0.45 and 0.41 under the BAU and RUD scenarios, respectively. Soil conservation was predicted to increase from 2.47 × 10^9^ tons in 2015 to 2.49 × 10^9^ tons in 2030 under the ELP scenarios, while the BAU and RUD scenarios were expected to remain stable from 2015 to 2030. Habitat quality was predicted to increase from 0.92 in 2015 to 0.93 in 2030 under the BAU and ELP scenarios, respectively, but decrease by 3% under the RUD scenario (0.89). The water purification was predicted to decrease slightly from 2015 to 2030 by 5% under the ELP and RUD scenarios, but decrease dramatically by 14% under the BAU scenario. The crop provision was predicted to remain stable from 2015 to 2030 under the RUD scenario but decrease by an even larger 30% under the BAU scenario. 

Forest and shrub land accounted for the largest proportion in carbon storage, soil conservation, and habitat quality in the ECA from 2000 to 2030 under three different scenarios (Tables S11–S15). Among them, the carbon storage and soil conservation in forest land were expected to increase from 0.605 × 10^8^ tons and 1.260 × 10^9^ tons in 2000 to 0.668 × 10^8^ tons and 1.410 × 10^9^ tons in 2030 under the ELP scenario, respectively. In 2000, the forest and shrub land presented the highest proportion in habitat quality, while it would be the lowest in 2030 under the ELP scenario. The cultivated land was the major source for nitrogen loading from 2000 to 2030, especially in 2000. In addition, 65% of the nutrients were exported from cultivated land during the same period. The water body presents the strongest capacity in regulating runoff, with an average value of 1 during the period from 2000 to 2030. Simultaneously, the ability of forest and shrub land to regulate runoff only changed somewhat, which were both no less than 0.35.

### 3.3. Changes in SES in the ECA From 2000 to 2030

**Figure 5:**
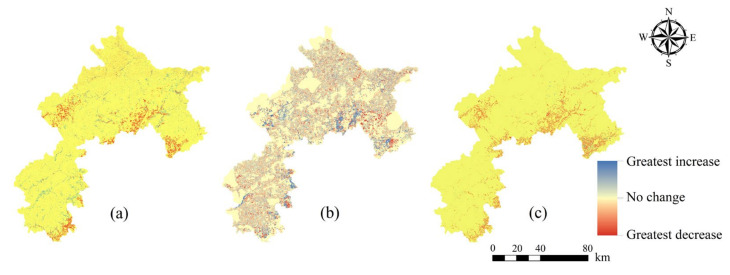
The spatial distribution of sum of ecosystem services (SES) changes from 2015 to 2030 under three alternative development scenarios: (**a**) 2015-BAU scenario, (**b**) 2015-ELP scenario, and (**c**) 2015-RUD scenario. The spatial patterns of SES during the years 2000–2030 are in Figures S1–S5.

During the period from 2000 to 2015, due to the increase of forest land, the sum of ecosystem services (SES) in the ECA showed an overall increasing trend with average values in 2000 and 2015 of 0.358 and 0.366, respectively (Figure 5). These changes will continue from 2015 to 2030 under three alternative scenarios, but there will be significant differences. Specifically, due to the limited urban expansion and implementation of ecological protection program envisioned by the ELP scenario, it is expected that the SES will increase to a greater extent and be strengthened in almost the whole region of ECA (0.378). Although water purification and crop provision will decline under the ELP scenario, other ecosystem services, such as carbon storage, flood regulation, soil conservation, and habitat quality, will perform better. The SES will decrease to a larger extent in space and intensity under the RUD scenario than it would under the BAU scenario (0.361 and 0.365, respectively), as the RUD scenario owning high proportions of built-up land is predicted to result in poor performance in most ES that are important.

### 3.4. Trade-Offs and Synergies Among Ecosystem Services 

**Figure 6:**
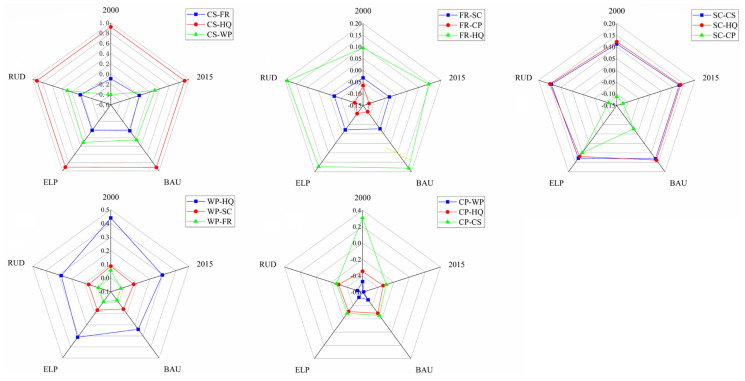
Radar plot of correlation coefficients among ESs in 2000–2030 under different scenarios. (CS-carbon storage, FR-flood regulation, SC-soil conservation, WP-water purification, HQ-habitat quality, and CP-crop provision).

There will be a significant (*p* < 0.05) correlation between carbon storage, soil conservation, water purification, habitat quality, and crop provision in the ECA during the period from 2000 to 2030, indicating trade-offs or synergies among these ecosystem services (Figure 6 and Table S16). Specifically, during the entire study period, carbon storage, soil conservation, and habitat quality are positively correlated with each other. These results indicate that there is a synergistic relationship between carbon storage, soil conservation, and habitat quality. The correlation between carbon storage and habitat quality is the strongest (r > 0.9), which could be explained by forest land that plays an important role in carbon sequestration and improving habitat quality. Carbon storage, habitat quality, and water purification are negatively correlated with crop production from 2015 to 2030 under three different scenarios, which indicates that there is a synergistic relationship of carbon storage, habitat quality, and water purification, and there is a trade-off relationship between them and crop production. At the same time, it also suggests that cultivated land has an intensive nutrient load. Flood regulation had a significant positive correlation with habitat quality (*p* < 0.001), but a significant negative correlation with crop provision (*p* < 0.01). There is no clear or weak relationship between flood regulation and most ES, as it was mainly affected by precipitation.

Generally speaking, during the whole study period, the relationship between pairs of the ecosystem service were consistent, but there were some exceptions. In 2000, there was a positive correlation between crop provision and carbon storage, while the relationship showed the opposite tendency from 2015–2030 under three different scenarios. This implies that cultivated land will have lower potential capacity for sequestering carbon in the future, as urban land continues to expand. In addition, in 2000, there was a negative correlation between water purification and carbon storage, but these two kinds of ES showed a positive correlation from 2015 to 2030 under three different scenarios, mainly due to the transition of cultivated land to forest and shrub land. The results also indicate that forest plays an important role in purifying pollutants.

## 4. Discussion

### 4.1. Impacts of Land-Use Change on Ecosystem Services

In the past two decades, the population growth, the rapid development of technology, and the real estate industry, and the government’s overall planning policies have aggravated land-use changes in Beijing. It has been demonstrated that land-use change has been identified as one of the most important driving factors of ecological change [2,56,57], and, ultimately, is the factor that determines the relative supply and strength of ES, and their interdependent processes [4,12,58]. In this study, the expansion of human-built landscapes resulted in the reduction of forest land, water bodies, and cultivated land, which, in turn, reduced the provision of multiple ES and changed the relationships between them as projected under three different scenarios. For example, 130.86 km^2^ of cultivated land is expected to be converted to built-up land, causing 0.13 × 10^7^ t decrease in carbon storage and 0.86 × 10^6^ t increase in soil conservation, respectively (Table S17). These results are consistent with the previous research conducted by [59,60]. At the same time, loss of agricultural area may lead to the loss of biodiversity (including native and wild cultivated species), ecological knowledge, and cultural infrastructure (such as irrigation channels) related to the ecosystem [61]. We also find, as previously reported, that human-built landscapes have a stronger capacity to reduce soil erosion because the impervious layer of concrete can prevent soil from being exposed on the surface [10,19,62]. However, increasing the area of the impervious layer to improve soil conservation service will inevitably lead to the loss of many biological properties of soil and trade-offs between soil conservation and other services, resulting in a decline in the sum of ecosystem services (SES). Under the RUD scenario, our study showed that soil conservation has a significant negative correlation with food production. Therefore, although urban expansion may promote regional economic advancement and provide shelter for human beings, the damage it brings to regulating and provisioning services cannot be ignored. Moreover, our results showed that there exist synergistic relationships between soil conservation and carbon storage, water purification, and habitat quality during the entire study period, which is contrary to the results of [12,63]. The different result may be due to the spatial heterogeneity of the ecosystem and the leading role of forest land in regulating and supporting ecological functions.

Compared with forest ecosystems, other ecosystems such as grassland ecosystems have less capacity to regulate rainfall and climate change due to lower plant density, carbon density, and root depth, and are prone to geological disasters such as landslides, debris flows, and water erosion [12,64]. In our study, forest land has the highest capacity in regulating flood and climate and conserving soil, followed by shrub land and grassland in 2030 under all three scenarios. Therefore, the implementation of the ecological protection plan under the ELP scenario is expected to restrict the human disturbance on reservoirs and 21 nature reserves, and promote vegetation regeneration in which the latter is the main factor determining the synergistic relationships among ES, resulting in the maximum SES (0.378). Our results also show that the above-mentioned policies have affected land-use changes and increased the expected provision of ES, but, at the cost of others, which inevitably lead to trade-offs among services. For example, the area of forest land is expected to increase by over 150 km^2^ under the ELP scenario, which leads to significant increases in carbon storage (0.13 × 10^7^ t), water purification (0.38 × 10^2^ t), and soil conservation (0.129 × 10^9^ t), relative to the baseline year. However, the expansion of forest land leads to the decline in food production, which is critical for the sustainable development of the ECA.

As an area that experienced dramatic social and economic transitions in Beijing, the sustainable development of the ECA requires adequate food supply sourced from local farmland. However, crop production was projected to decrease from 2000 to 2030, except for under the RUD scenario, which was expected to remain relatively stable. The reason for this projected stability is that urbanization will inevitably occupy a large amount of cultivated land [19], and the specific policy advocates returning cultivated land to forest. In addition, although population growth will inevitably have a huge demand for food under the RUD scenario, well-developed transportation and technology can facilitate food being imported to Beijing. Therefore, coordinating economic development and the protection of natural ecosystems requires further research.

### 4.2. Strategies and Implications

Optimal land-use management needs to comprehensively consider the possible results of all the scenarios, and integrate ecosystem services assessments into land-use planning and management. While managing complexity is always a challenge, the ecological conservation area (ECA) in Beijing is an important ecological reservoir and water source protection area facing serious habitat degradation, which urgently requires our attention. Our study results showed that, in the past 15 years, the ecosystem structure of the ECA has undergone significant changes due to the influence of human activities, the frequent occurrence of natural disasters, and the implementation of a series of policies (such as ecological red lining and basic farmland protection). Based on the potential changes of land-use, we proposed two land-use scenarios to provide a framework for future development in the ECA, rather than focusing on the single prediction result. Of these, the RUD scenario is the maximization of urban development, while the ELP scenario is the maximization of ecological benefits. Compared with these two scenarios, the BAU scenario inherited the trend of land-use change from 2000 to 2015. Therefore, we suggest that the policies for maintaining and enhancing ecosystem services under the ELP scenario will be our first choice in the future because the SES reaches the highest value.

For sustainable land-use and the improvement of ecosystem services for the ECA, we propose several strategies as management guidelines: (1) implementation of ecological environment construction [27,65,66], such as: (i) Ecological Restoration and Management of Abandoned Mines in the western mountains, (ii) Conversion of Farmland to Forests and Ecological Compensation, and (iii) Ecological Protection Red Line in Beijing. (2) Optimizing land-use structure and implementing comprehensive development strategies: (i) Overall planning for the urban and rural human-built landscapes, and promotion of efficient governance [65,67], (ii) optimize the structure of human-built landscapes, production, living, and ecological land, such as improving the vertical utilization rate and redeveloping discarded factories [68], while increasing urban green space to mitigate the negative effects of urban expansion on ecosystem services, (iii) build a new ecological-oriented comprehensive development mode, including comprehensive management of soil erosion, and ecological tourism in mountainous areas [69], and (iv) strictly control development activities of core areas of water source protection areas, encourage ecological relocation, increase ecological restoration, and carry out ecological construction of watersheds [27,29]. In conclusion, only through rational land-use planning, the harmonious development of natural ecosystems and socio-economic systems can be realized to meet current and future needs.

### 4.3. Limitations and Future Perspectives

The main focus of our study was simulating land-use change and the resulting changes in ecosystem services, linking the FLUS and InVEST models to assess the impacts of land-use on ES and their trade-offs [11,70,71]. The FoM value of simulation results was 0.269, and the value of ecosystem services was similar to that of [25], indicating that the proposed models are highly applicable for assessing the effects of land-use on ES in the ECA. Land-use simulation and ecosystem services assessments can provide valuable information for researchers and policymakers to better understand trade-offs, draft appropriate measures, and to develop informed strategies to better adapt to different future scenarios [72]. However, in most cases, there are still some limitations in simulation of land-use. For example, the transition rules of the GeoSOS-FLUS model remain unchanged, but will actually change in the future—whether it is in 50 or 100 years [20,73]. Moreover, these three alternative land-use scenarios cannot represent all the possibilities that occur in the future, as climate change scenarios and political implications are not incorporated into the future LUCC simulations.

We also recognize some data limitations in the InVEST model. First, this model assumes that none of the land-use types in the landscape are gaining or losing carbon over time. According to this hypothesis, the only changes of carbon storage over time are due to changes from one land-use type to another. However, many areas are recovering from the land-use in the past, or are undergoing natural succession. Second, the “Annual Water Yield” model does not consider the complex spatial distribution of land-use, which may induce complex water balances, may not be well captured by the model. Third, the “Water Purification” model does not involve any chemical or biological interactions from the pollution point to the load except for filtration by vegetation. Therefore, through interaction with air, water, other pollutants, bacteria, or other factors, the nutrient retention capacity may be reduced [47].

In addition, there are several imperfections in the index for the sum of ecosystem services (SES) in the ECA. First, the SES does not fully reflect the overall level of ES in this study, as social and cultural services (e.g., recreation and tourism) are not considered, which is mainly due to the lack of local population data and the imperfect existing quantitative methods. Second, the weights of the ES calculated for the SES index are subjective, even though they partially reflect reality, which is something we need to improve in the future. Third, this study did not take into account the possible constraints, such as investment and cost of implementing land-use strategy. In the future, we should spare no effort to deal with these challenges, especially the significant effects of future climate changes. Therefore, the background climate conditions under the future land-use scenarios cannot be ignored. 

## 5. Conclusions

Based on the InVEST and GeoSOS-FLUS models, this study explored how land-use changes would alter multiple ecosystem services and their trade-offs under three different alternative scenarios in the ECA. According to our estimates, the built-up land, forest, and shrub land have increased in varying degrees in the past 15 years. The forest land was predicted to increase the most from 2015 to 2030 under the ELP scenario, while the area of built-up land was predicted to increase by 0.96% and 2.52% under the BAU and RUD scenarios, respectively. Therefore, the ELP scenario would present the highest level in carbon storage, habitat quality, soil conservation, and flood regulation, resulting in the highest of the sum of ecosystem services (SES). Compared with the BAU scenario, the SES under the RUD scenario would decrease to a larger extent. We recommend the ELP scenario as our mode for promoting sustainability in the ECA. Based on these findings, we propose several strategies, including ecological environment construction and comprehensive development in the ECA to achieve goals of sustainable development.

## Figures and Tables

**Table 1:** Spatial data used in the InVEST model.

Types	Description	Sources
Land-use/cover	Land-use maps in 2000 and 2015 (30 m)	Resources and Environmental Sciences, Chinese Academy of Sciences [32]
DEM	Digital Elevation Model (30 m)	Resources and Environmental Sciences, Chinese Academy of Sciences [32]
Climate data	Annual precipitation, monthly precipitation, temperature, sunshine hours (1 km)	National Earth System Science Data Center [33]
Soil data	Soil texture, sand fraction, silt fraction, clay fraction, root restricting layer depth, plant available water content (1 km)	Harmonized World Soil Database [34]
Evapotranspiration coefficient (*K_c_*)	Evapotranspiration for reference crops	Food and Agriculture Organization of the United Nations [35]
Soil erodibility (K)	A soil erodibility value for each cell (1 km)	National Earth System Science Data Center [33]
Rainfall erosivity (R)	An erosivity index value for each cell (1 km)	National Earth System Science Data Center [33]
Potential evapotranspiration (ET0)	Plant evapotranspiration (1 km)	Global Aridity Index and Potential Evapo-Transpiration (ET0) Climate Database v2 [36]
Sub-watersheds	Each watershed contributes to a point of interest	HydroSHEDS [37]
Carbon pools	Four basic carbon densities for each land cover type	[25,28,38]

**Table 2:** Descriptions of alternative scenarios and their land cover changes.

Scenario	Description	Major Land Cover Changes
BAU (Business As Usual) scenario	The land-use pattern is only affected by the historical transition rules and simulated without any constraints.	Built-up land would continue to expand from 2015 to 2030 and occupy both cultivated and ecological land.
ELP (Ecological Protection) scenario	According to General Planning of Beijing Municipality (2016–2035) and related ecological construction policies, natural reserves, reservoirs, and basic farmland are constraints.	An increase in built-up land and forest land relative to the baseline case scenario. Cultivated land would account for more than 7% of the study area.
RUD (Rapid Urban Development) scenario	Due to the rapid increases of population and technologies, the demands for built-up land, including urban and rural residential land, construction land, and transport facility areas would expand rapidly. Basic farmland is prevented from changing to the other.	An increase in built-up land and a slight decrease in cultivated land, respectively, relative to the baseline case scenario.

**Table 3:** Index weights of each ecosystem service (ES) for the ecological conservation area.

Indicators	Carbon Storage	Flood Regulation	Water Purification	Soil Conservation	Habitat Quality	Crop Production
Weight	0.1596	0.1688	0.0875	0.1396	0.1574	0.0964

**Table 4:** The supply of multiple ecosystem service (ES) from 2000 to 2015 and the projected ES from 2015 to 2030 under three different scenarios.

Indicators	Carbon Storage (10^8^ t)	Flood Regulation (dimensionless)	Soil Conservation (10^9^ t)	Habitat Quality (dimensionless)	Water Purification (10^3^ t)	Crop Provision (10^6^ t)
2000	0.95	0.39	2.69	0.86	1.91	0.57
2015	0.99	0.53	2.47	0.92	1.39	0.47
BAU	0.99	0.45	2.47	0.93	1.20	0.32
ELP	1.00	0.56	2.49	0.93	1.32	0.46
RUD	0.97	0.41	2.47	0.89	1.33	0.47

## Supplementary Materials

The following are available online at https://www.mdpi.com/1660-4601/17/22/8632/s1. Figure S1: The spatial distribution of the sum of total ecosystem services (SES) in 2000. Figure S2: The spatial distribution of the sum of total ecosystem services (SES) in 2015. Figure S3: The spatial distribution of the sum of total ecosystem services (SES) under the BAU scenario. Figure S4: The spatial distribution of the sum of total ecosystem services (SES) under the ELP scenario. Figure S5: The spatial distribution of the sum of total ecosystem services (SES) under the RUD scenario. Table S1: Description of land use types. Table S2: Driving factors of land use simulation. Table S3: Input data on carbon stoRUD in each of the four fundamental pools for each LULC class in the InVEST 3.8.0 Carbon Storage and Sequestration model (Mg/ha). Table S4: Input data for each LULC class in the InVEST 3.8.0 Annual Water Yield model. Table S5: Input data for each LULC class in the InVEST 3.8.0 Sediment Delivery Ratio model. Table S6: Input data for each LULC class in the InVEST 3.8.0 Nutrient Delivery Ratio model. Table S7: Sensitivity of land cover classes to each threat in the InVEST 3.8.0 habitat quality model. Table S8: Threats data for each LULC class in the InVEST 3.8.0 Habitat Quality model. Table S9: Land-use area (km^2^) and percent area (%) for each land-use type from 2000 to 2030 under the BAU, ELP, and RUD scenarios in the ecological conservation area. Table S10: Conversion cost matrix from 2000 to 2015 (km^2^). Table S11: Carbon storage (CS) for each land-use type from 2000 to 2030 under the BAU, ELP, and RUD scenarios (10^8^ tons). Table S12: Flood regulation (FR) for each land-use type from 2000 to 2030 under the BAU, ELP, and RUD scenarios (Unitless). Table S13: Soil conservation (SC) for each land-use type from 2000 to 2030 under the BAU, ELP, and RUD scenarios (10^9^ tons). Table S14: Water purification (WP) for each land-use type from 2000 to 2030 under the BAU, ELP, and RUD scenarios (10^3^ tons). Table S15: Habitat quality (HQ) for each land-use type from 2000 to 2030 under the BAU, ELP, and RUD scenarios (Unitless). Table S16: Correlation among six ecosystem services for 2000 and 2030 under each scenario. Table S17: Ecosystem service (ES) change matrix driven by per-unit land use transitions of the main land use types from 2015 to 2030 under the BAU, ELP, and RUD scenarios.
