# Supplementary material for: Analyzing Land-Use Change Scenarios for Ecosystem Services and their Trade-Offs in the Ecological Conservation Area in Beijing, China
Source: Int J Environ Res Public Health. 2020 Nov 20;17(22):8632. doi: 10.3390/ijerph17228632 (PMC7699891; doi:10.3390/ijerph17228632)
Supplement: Supplementary file 1 [file ijerph-17-08632-s001.pdf]

Supplementary material

Analyzing land-use change scenarios for ecosystem services and their trade-offs in the ecological conservation area in Beijing, China

Methods

Land-use demand projection

Markov model is a non-spatial demand model of land-use types in the future, which is used to calculate the transition probability of land use types over time in this study (Nor et al., 2017). The land-use maps of different time intervals were derived from simulations and compared with each other in matrix according to the maximum probability value (Nor et al., 2017). Then, the maximum probability of each grid cell that either remained unchanged or was transformed into another class was calculated. Finally, the Markov model was applied using the probability transition matrix and transition maps of each type to another type under the following equation:

$$S_{ij}(t+1) = P_{ij}S_i(t) \quad (1)$$

where $S_{ij}(t+1)$ and $S_i(t)$ are the status of land-use type i at the time of t+1 and t; P_{ij} is the transition probability matrix of land-use type i to j.

Supplementary Figures

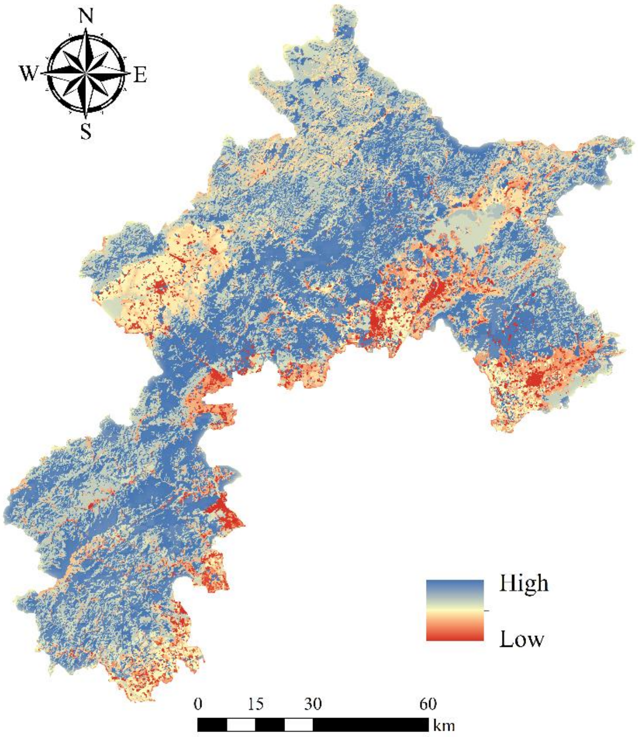

Figure S1. The spatial distribution of the sum of total ecosystem services (SES) in 2000.

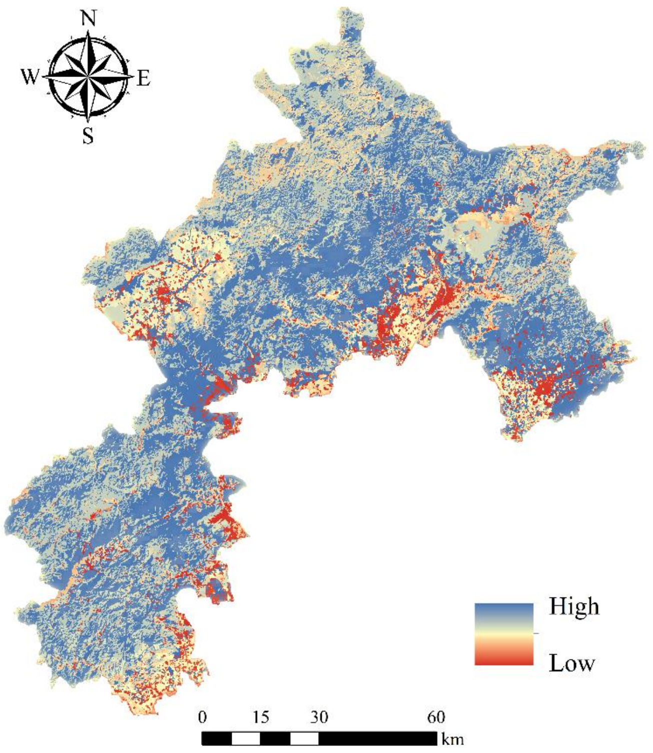

Figure S2. The spatial distribution of the sum of total ecosystem services (SES) in 2015.

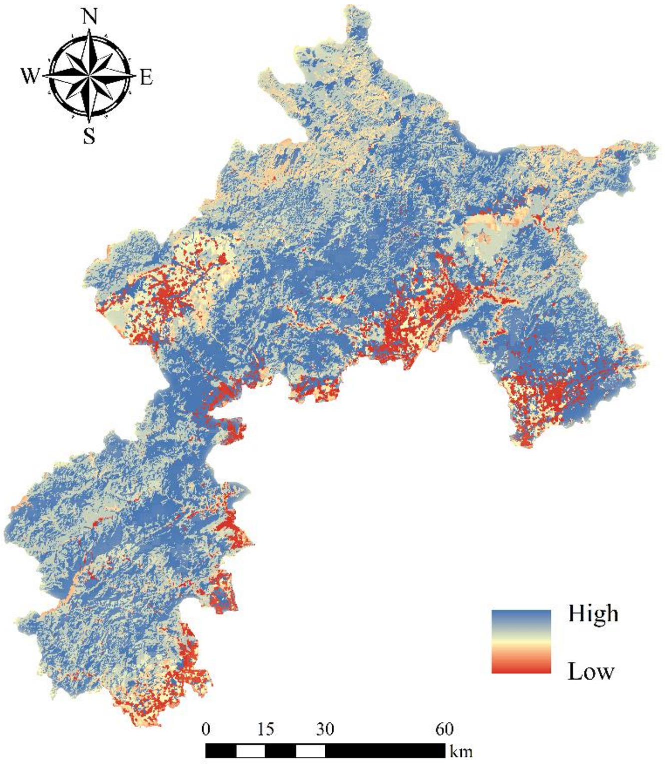

Figure S3. The spatial distribution of the sum of total ecosystem services (SES) under the BAU scenario.

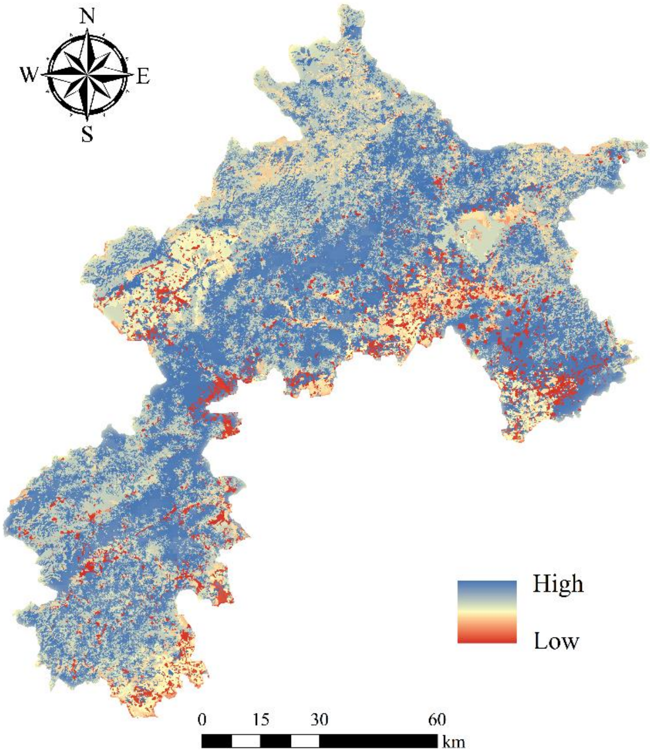

Figure S4. The spatial distribution of the sum of total ecosystem services (SES) under the ELP scenario.

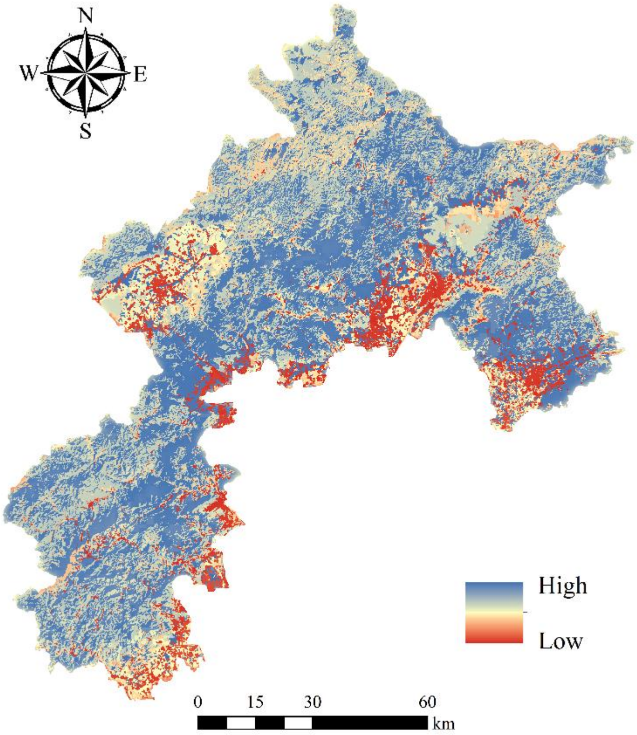

Figure S5. The spatial distribution of the sum of total ecosystem services (SES) under the RUD scenario.

Supplementary Tables

Table S1. Description of land use types.

Type	Description
Forest land (FL)	Evergreen coniferous forests, deciduous coniferous forests, deciduous broad-leaf forests, and mixed broadleaf conifer forests
Built-up land (BL)	Urban and rural settlements, commercial areas, industrial areas, construction areas, and transport facility areas
Shrub land (SL)	Mix of small trees (< 5m tall) and other natural covers
Cultivated land (CL)	Irrigated and dry croplands, orchards
Grass land (GL)	Mainly grass fields (dense, moderate, and low coverage grasses)
Water body (WB)	Rivers, lake/ponds, canals, and reservoirs
Unused land (UL)	Cliffs/small landslide, bare rocks, other permanently abandoned rock stony land, sand areas, other unutilized lands

Table S2. Driving factors of land use simulation.

Data	Year	Resolution	Data resource
**DEM**	2000	30 m	Resources and Environmental Data Cloud Platform (http://www.resdc.cn/)
**Slope**	2000	30 m	Calculated from DEM
**Aspect**	2000	30 m	Calculated from DEM
**Relief amplitude**	2000	1 km	National Earth System Science Data Center (http://www.geodata.cn/)
**GDP**	2000, 2015	1 km	Resources and Environmental Data Cloud Platform (http://www.resdc.cn/)
**Population**	2000, 2015	1 km	Resources and Environmental Data Cloud Platform (http://www.resdc.cn/)
**Annual precipitation**	2015	1 km	National Earth System Science Data Center (http://www.geodata.cn/)
**Annual mean temperature**	2015	1 km	National Earth System Science Data Center (http://www.geodata.cn/)
**Distance from road**	2017	1 km	National Geomatic Center of China (http://www.ngcc.cn/)
**Distance from residential area**	2017	1 km	National Geomatic Center of China (http://www.ngcc.cn/)
**Traffic station**	2017	1 km	National Geomatic Center of China (http://www.ngcc.cn/)
**Distance from river**	2017	1 km	National Geomatic Center of China (http://www.ngcc.cn/)
**Distance from landslide and collapse point**	2015	1 km	China Geological Survey (http://www.cgs.gov.cn/)
**Soil attributes**	2012	1 km	Harmonized World Soil Database (http://webarchive.iiasa.ac.at/Research/LUC/External-World-soil-database/)

Table S3. Input data on carbon stoRUD in each of the four fundamental pools for each LULC class in the InVEST 3.8.0 Carbon Storage and Sequestration model (Mg/ha).

Land use/land cover type	Aboveground biomass	Belowground biomass	Soil	Dead organic matter
Grassland	3.46	0	30.17	0
Water body	0	0	0	0
Cultivated land	17.29	0	22.5	0
Built–up land	0	0	0	0
Unused land	0	0	0	0
Forest land	24.45	4.51	93.85	12.53
Shrub land	5.72	0.36	63.05	3.49

Table S4. Input data for each LULC class in the InVEST 3.8.0 Annual Water Yield model.

Land use/land cover type	Max root depth (mm)	Evapotranspiration coefficient
Grassland	1400	0.591
Water body	0	1
Cultivated land	1500	0.683
Built–up land	0	0.3
Unused land	0	0.2
Forest land	3000	0.9
Shrub land	2000	0.5

Table S5. Input data for each LULC class in the InVEST 3.8.0 Sediment Delivery Ratio model.

Land use/land cover type	Cover and management factor	management practice factor
Grassland	0.01	0.2
Water body	0.001	0.001
Cultivated land	0.5	0.4
Built–up land	0.001	0.001
Unused land	0.25	0.01
Forest land	0.003	0.2
Shrub land	0.01	0.2

Table S6. Input data for each LULC class in the InVEST 3.8.0 Nutrient Delivery Ratio model.

Land use/land cover type	lucode	Kc	Root depth	Load n	Eff n	Crit len n	Proportion subsurface n
Grassland	1	0.5	2000	6.26	0.65	150	0
Water body	2	1.2	1	0	0	0	0
Cultivated land	3	0.65	1700	22.8	0.35	25	0.5
Built–up land	4	0	1	5.51	0.01	10	0
Unused land	5	0	1	0.001	0.05	10	0
Forest land	6	0.9	7000	3.88	0.8	300	0
Shrub land	7	0.8	4500	5.05	0.65	230	0

Table S7. Sensitivity of land cover classes to each threat in the InVEST 3.8.0 habitat quality model.

Land use/land cover type	HABITAT	L crp	L urb	L wat
Grassland	0.6	0.3	0.6	0.3
Water body	0.5	0.2	0.7	0
Cultivated land	0.4	0	0.4	0.1
Built–up land	0	0	0	0
Unused land	0	0	0	0
Forest land	1	0.4	0.85	0.4
Shrub land	0.7	0.3	0.6	0.2

Table S8. Threats data for each LULC class in the InVEST 3.8.0 Habitat Quality model.

THREAT	MAX DIST	WEIGHT	DECAY
crp	2	0.55	linear
urb	4	1	linear
wat	1	0.3	linear

Table S9. Land-use area (km^2) and percent area (%) for each land-use type from 2000 to 2030 under the BAU, ELP, and RUD scenarios in the ecological conservation area.

Types	2000 (km^2/%)	2015 (km^2/%)	BAU (km^2/%)	ELP (km^2/%)	RUD (km^2/%)
Grassland	662.64 (5.95)	793.74 (7.12)	866.58 (7.78)	751.48 (6.74)	791.36 (7.10)
Water body	258.36 (2.32)	195.19 (1.75)	142.92 (1.28)	174.64 (1.57)	179.37 (1.61)
Cultivated land	1688.09 (15.15)	931.22 (8.36)	718.01 (6.44)	849.83 (7.63)	799.27 (7.17)
Built–up land	508.26 (4.56)	710.49 (6.38)	817.75 (7.34)	730.83 (6.56)	991.67 (8.90)
Unused land	29.46 (0.26)	18.28 (0.16)	15.24 (0.14)	13.43 (0.12)	8.83 (0.08)
Forest land	4468.45 (40.10)	4918.48 (44.14)	4995.83 (44.83)	5070.86 (45.50)	4798.94 (43.06)
Shrub land	3529.30 (31.67)	3576.73 (32.10)	3587.88 (32.20)	3552.12 (31.87)	3574.94 (32.08)

Table S10. Conversion cost matrix from 2000 to 2015 (km^2).

Land use types	Grass land	Water body	Cultivated land	Built-up land	Unused land	Forest land	Shrub land	Total
Grass land	488.63	26.05	186.49	8.30	5.54	25.99	52.62	793.63
Water body	1.09	169.42	16.51	1.19	0.30	4.41	2.27	195.18
Cultivated land	21.70	34.07	812.64	16.78	2.64	18.19	25.23	931.25
Built-up land	18.61	4.69	170.06	462.87	6.02	20.51	27.70	710.47
Unused land	1.28	0.42	3.99	0.31	9.72	0.92	1.64	18.28
Forest land	60.48	9.43	413.65	10.71	3.27	4122.92	297.63	4918.09
Shrub land	70.77	14.24	84.49	8.09	1.97	275.15	3121.83	3576.54
Total	662.58	258.32	1687.83	508.24	29.46	4468.09	3528.92	11143.45

Table S11. Carbon storage (CS) for each land-use type from 2000 to 2030 under the BAU, ELP, and RUD scenarios (10^8 tons).

	Grassland	Water body	Cultivated land	Built–up land	Unused land	Forest land	Shrub land
2000	0.022	0	0.067	0	0	0.605	0.256
2015	0.027	0	0.038	0	0	0.655	0.264
BAU	0.030	0	0.027	0	0	0.654	0.267
ELP	0.025	0	0.035	0	0	0.668	0.267
RUD	0.027	0	0.032	0	0	0.638	0.267

Table S12. Flood regulation (FR) for each land-use type from 2000 to 2030 under the BAU, ELP, and RUD scenarios (Unitless).

	Grassland	Water body	Cultivated land	Built–up land	Unused land	Forest land	Shrub land
2000	0.26	1	0.30	0.23	0.09	0.39	0.35
2015	0.26	1	0.31	0.23	0.08	0.39	0.35
BAU	0.28	1	0.31	0.25	0.13	0.38	0.35
ELP	0.26	1	0.31	0.22	0.09	0.39	0.35
RUD	0.26	1	0.31	0.24	0.09	0.38	0.35

Table S13. Soil conservation (SC) for each land-use type from 2000 to 2030 under the BAU, ELP, and RUD scenarios (10^9 tons).

	Grassland	Water body	Cultivated land	Built–up land	Unused land	Forest land	Shrub land
2000	0.153	0.009	0.100	0.034	0.007	1.260	1.126
2015	0.111	0.007	0.027	0.063	0.001	1.281	0.971
BAU	0.115	0.004	0.023	0.056	0.001	1.289	0.973
ELP	0.090	0.004	0.024	0.088	0.001	1.410	0.879
RUD	0.111	0.006	0.025	0.075	0.001	1.274	0.988

Table 14. Water purification (WP) for each land-use type from 2000 to 2030 under the BAU, ELP, and RUD scenarios (10^3 tons).

	Grassland	Water body	Cultivated land	Built–up land	Unused land	Forest land	Shrub land
2000	0.065	0.058	1.243	0.076	0.003	0.188	0.272
2015	0.098	0.052	0.650	0.089	0.001	0.220	0.262
BAU	0.130	0.040	0.410	0.112	0.001	0.244	0.259
ELP	0.083	0.042	0.578	0.094	0.001	0.258	0.253
RUD	0.098	0.045	0.559	0.145	0.002	0.213	0.265

Table S15. Habitat quality (HQ) for each land-use type from 2000 to 2030 under the BAU, ELP, and RUD scenarios (Unitless).

	Grassland	Water body	Cultivated land	Built–up land	Unused land	Forest land	Shrub land
2000	0.588	0.490	0.397	0	0	0.974	0.686
2015	0.585	0.497	0.401	0	0	0.961	0.692
BAU	0.582	0.480	0.374	0	0	0.948	0.697
ELP	0.586	0.496	0.408	0	0	0.953	0.698
RUD	0.585	0.496	0.400	0	0	0.959	0.696

Table 16. Correlation among six ecosystem services for 2000 and 2030 under each scenario.

Scenarios	Block samples	Ecosystem services					
	N=2000	Carbon storage	Flood regulation	Soil conservation	Water purification	Habitat quality	Crop provision
2000	Carbon storage	1	-0.093**	0.113**	-0.404**	0.918**	0.309**
	Flood regulation		1	-0.032	0.057*	0.096**	-0.065**
	Soil conservation			1	0.087**	0.122**	-0.112**
	Water purification				1	0.440**	-0.470**
	Habitat quality					1	-0.344**
	Crop provision						1
BAU	Carbon storage	1	0.030	0.131**	0.251**	0.912**	-0.251**
	Flood regulation		1	-0.027	-0.023	0.181**	-0.117**
	Soil conservation			1	0.057*	0.138**	-0.027
	Water purification				1	0.240**	-0.486**
	Habitat quality					1	-0.283**
	Crop provision						1
ELP	Carbon storage	1	0.018	0.128**	0.309**	0.907**	-0.284**
	Flood regulation		1	-0.021	-0.010	0.173**	-0.107**
	Soil conservation			1	0.067*	0.119**	0.100**
	Water purification				1	0.312**	-0.524**
	Habitat quality					1	-0.310**
	Crop provision						1
RUD	Carbon storage	1	0.024	0.143**	0.287**	0.915**	-0.266**
	Flood regulation		1	-0.021	-0.006	0.189**	-0.113**
	Soil conservation			1	0.070*	0.149**	-0.114**
	Water purification				1	0.281**	-0.534**
	Habitat quality					1	-0.293**
	Crop provision						1

* $p < 0.05$.** $p < 0.001$.

Table S17. Ecosystem service (ES) change matrix driven by per-unit land use transitions of the main land use types from 2015 to 2030 under the BAU, ELP, and RUD scenarios.

	**Conversions**	**Area (km^2)**	**Carbon storage (10^4 Mg)**	**Water yield (10^6 m^3)**	**Soil conservation (10^5 t)**	**Water purification (10^3 t)**	**Habitat quality (Unitless)**
2015-BAU	CL to GL	48.98	0.34	0.27	1.19	-17.76	0.10
	CL to BL	133.99	-44.18	4.45	8.69	-67.46	-0.33
	CL to FL	39.49	0.09	-0.09	1.69	-5.59	-0.01
	FL to SL	78.56	-1.30	0.03	-0.56	0.01	-0.01
	SL to FL	77.04	1.25	-0.02	0.14	-0.09	0.01
2015-ELP	GL to FL	179.49	163.45	-8.74	96.46	-4.93	0.34
	CL to FL	100.19	85.71	-3.25	15.47	-54.79	0.51
	CL to SL	56.62	17.29	-1.06	8.28	-35.44	0.27
	BL to GL	132.36	43.43	-2.41	-24.79	-3.32	0.57
	BL to FL	82.39	107.55	-5.97	-0.12	-3.29	0.93
	FL to GL	102.89	-92.79	4.94	-47.29	2.57	-0.34
	FL to BL	164.25	-200.22	10.61	0.43	7.12	-0.87
	SL to GL	146.41	-48.76	4.89	-55.47	0.94	0.08
	SL to FL	1121.00	595.18	-14.55	305.56	-18.24	0.24
2015-RUD	CL to BL	133.95	-46.41	4.59	8.61	-65.13	-0.35
	FL to BL	130.86	-150.42	8.01	0.30	4.88	-0.81

References

Nor, A.N.M.; Corstanje, R.; Harris, J.A.; Brewer, T. Impact of rapid urban expansion on green space structure. *Ecol. Indic.* **2017**, *81*, 274–284, doi:10.1016/j.ecolind.2017.05.031.
